# Supplementary material for: MultipleXed Population Selection and Enrichment single nucleus RNA sequencing (XPoSE-seq) enables sample identity retention during transcriptional profiling of rare populations
Source: bioRxiv. 2023 Sep 29:2023.09.27.559834. Preprint. [Version 1] doi: 10.1101/2023.09.27.559834 (PMC10775316; doi:10.1101/2023.09.27.559834)
Supplement: 1 [file NIHPP2023.09.27.559834V1-supplement-1.pdf]

bioRxiv preprint doi: https://doi.org/10.1101/2023.09.27.559834; this version posted September 29, 2023. The copyright holder for this preprint (which was not certified by peer review) is the author/funder, who has granted bioRxiv a license to display the preprint in perpetuity. It is made available under aCC-BY 4.0 International license.

Supplementary Figures

A. mRFP timecourse after novel context

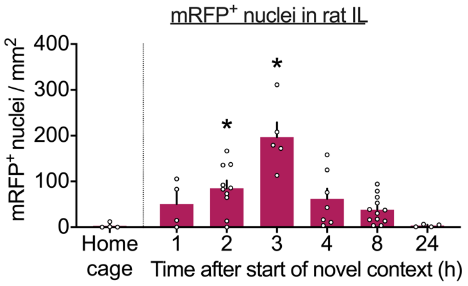

Figure S1. mRFP labels Fos-expressing active neurons in *Fos-mRFP* rats. **A**, Timecourse of mRFP labeling following 1 hour of novel context exploration in *Fos-mRFP* rats (n = 4 – 12 per group). Data is expressed as means ± S.E.M. * significant difference from homecage (HC). See Table S1 for detailed listing of all statistical outputs.

bioRxiv preprint doi: https://doi.org/10.1101/2023.09.27.559834; this version posted September 29, 2023. The copyright holder for this preprint (which was not certified by peer review) is the author/funder, who has granted bioRxiv a license to display the preprint in perpetuity. It is made available under aCC-BY 4.0 International license.

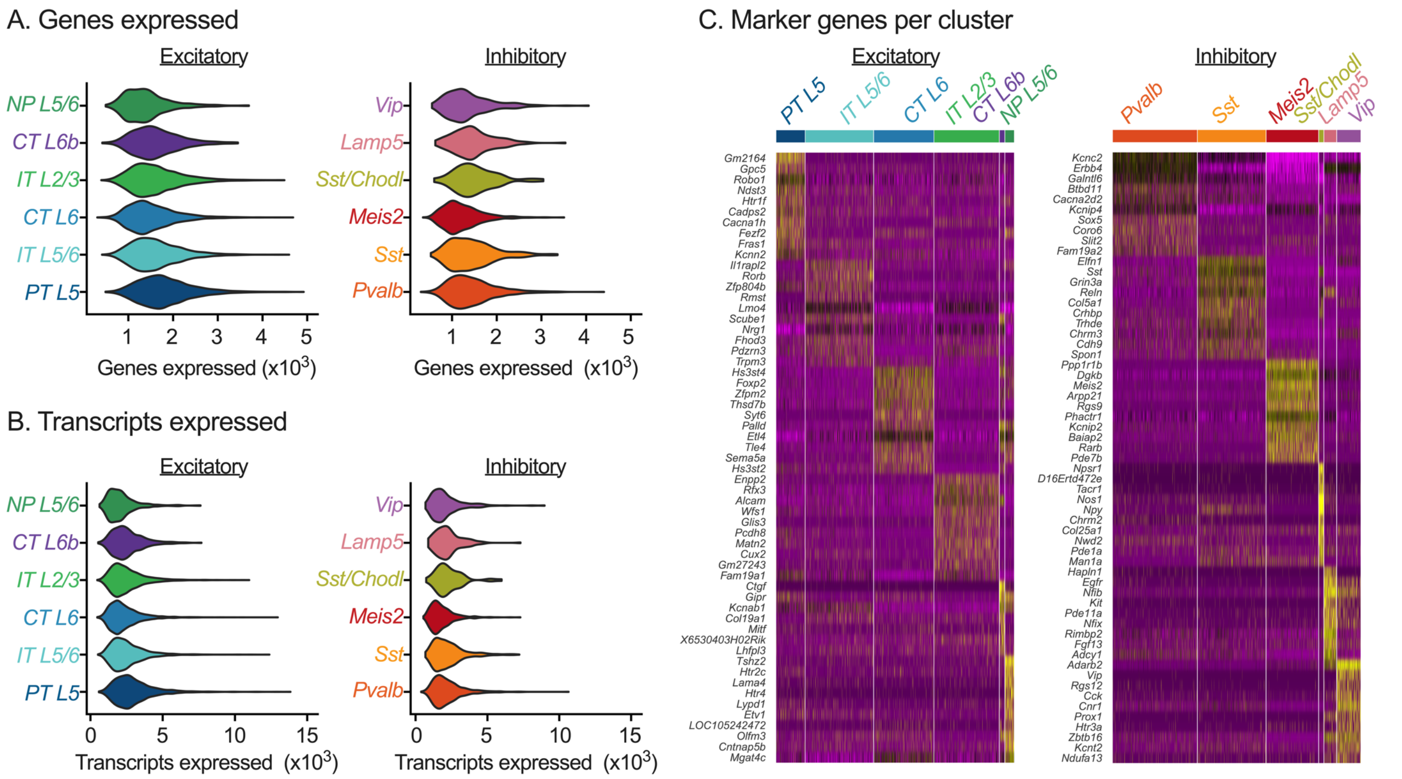

Figure S2. Quality control and cluster markers of excitatory and inhibitory neuron clusters. (**A**) Genes expressed in excitatory and inhibitory neuron clusters. Violin plot shows the number of genes expressed in excitatory (left) and inhibitory (right) cluster types collapsed across biological replicates and populations (n = 8). (**B**) Total number of transcripts expressed. Violin plot shows the total number of transcripts expressed in excitatory (left) and inhibitory (right) cluster types. (**C**) Heatmap of top ten genes expressed in major excitatory (left) and inhibitory (right) neuron clusters of the rat IL.

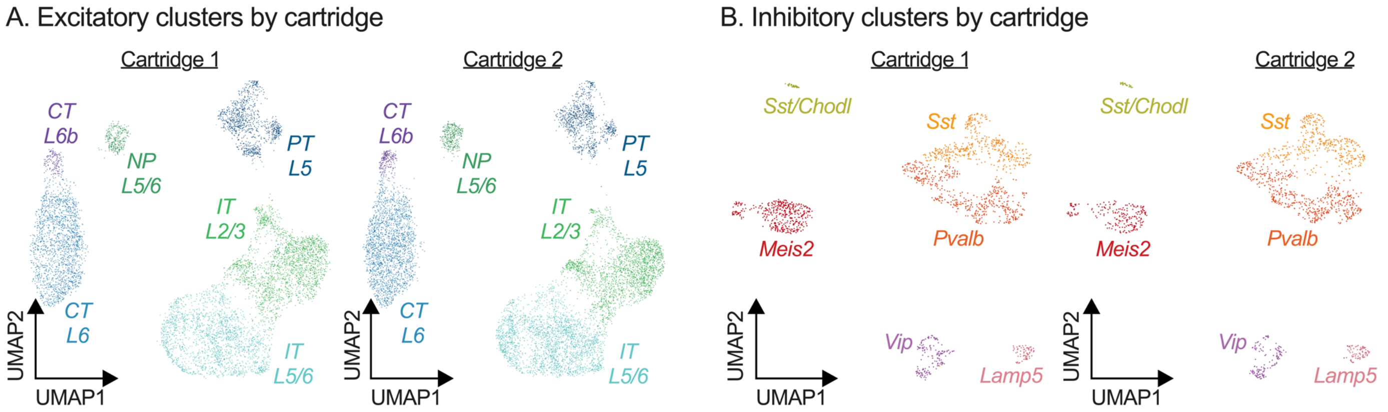

Figure S3. Neuronal cell type cluster type distribution between cartridges. (**A**) UMAP representation of excitatory neuron cluster distribution by cartridge. (**B**) UMAP representation of inhibitory neuron clusters by cartridge.
